# The Chromatin Remodeler Protein CHD4 Cooperates With NKX2.2 to Regulate Pancreatic Beta Cell Integrity

**DOI:** 10.1101/2025.06.16.659956

**Published:** 2025-06-17

**Authors:** Dylan K. Sarbaugh, Thais Gaia Oliveira, Michelle A. Guney, Victoria M. Hoelscher, Christopher J. Hill, Cole R. Michel, Kristen L. Wells, Richard K.P. Benninger, Lori Sussel

**Affiliations:** 1 Barbara Davis Center for Diabetes, University of Colorado Anschutz Medical Campus, Aurora, CO 80045; 2 School of Pharmacy, Mass Spectrometry Facility, University of Colorado Anschutz Medical Campus, Aurora, CO 80045

## Abstract

NKX2.2 is a transcription factor that regulates pancreatic islet beta (β) cell identity and function; however, cofactor proteins that modulate the functional activity of NKX2.2 in β cells are relatively unexplored. An unbiased proteomics screen identified chromodomain helicase DNA-binding protein 4 (CHD4) as an NKX2.2 interacting partner. CHD4 is a nucleosome remodeler that directs the appropriate differentiation, maturation and function of many cell types by activating or repressing genes. To characterize the NKX2.2-dependent and independent roles of CHD4 in β cells, we generated *Chd4* βKO mice. Deletion of *Chd4* substantially impaired the maturation and function of β cells. The *Chd4* βKO mice were diabetic as early as 3 weeks of age due to the disruption of islet integrity, impaired glucose-stimulated insulin secretion and calcium signaling, and downregulation of essential β cell regulatory genes. These studies demonstrate that CHD4 is an essential transcriptional cofactor of NKX2.2 that is required for the proper maturation and function of pancreatic β cells.

## Introduction

Blood glucose homeostasis is maintained by the pancreatic islets of Langerhans. Adult pancreatic islets are comprised of four major hormone producing endocrine cell types including the beta (β) cells that secrete insulin to lower blood glucose levels and pancreatic alpha (α) cells that secrete glucagon to raise blood glucose levels. When pancreatic β cells become dysfunctional or destroyed, diabetes mellitus occurs, which is an increasingly prevalent disease worldwide. Understanding the transcriptional networks and proteins that maintain appropriate pancreatic β cell maturation and function are essential for understanding the pathophysiology of diabetes to facilitate improved treatment options and/or leading to a cure for diabetes.

The combinatorial temporal and spatial expression of transcription factors allows for appropriate cell differentiation, maturation, and function. In the pancreas, numerous studies over the past 30 years have identified many of the transcriptional regulatory pathways that regulate islet cell development and function (1–3). Each islet population initiates independent transcriptional programs, expressing a cohort of essential transcription factors that help establish its proper development, maturation, and functional identity.

NKX2.2 is an essential transcription factor required for the proper development, maturation and function of β cells in mice and humans. In *Nkx2.2* null mice, there is a complete absence of β cells, a severe reduction of α cells, and a substantial increase in ghrelin producing cells, leading to neonatal diabetes and death (4, 5). In humans, homozygous loss of function mutations in *Nkx2.2* also results in neonatal diabetes (6). To study the role of NKX2.2 specifically in β cells, *Nkx2.2* was deleted in developing and mature β cells (*Nkx2.2* βKO mice). These mice survive into adulthood, however the mutant β cells were severely dysfunctional and a proportion of β cells either failed to produce insulin or lost their β cell identity and became polyhormonal (7).

NKX2.2 has 3 protein domains, the tinman (TN) domain, the homeodomain (HD) which binds DNA and the NK2 specific (SD) domain. To dissect which domains of NKX2.2 are important for β cell function, mouse models that deleted either the TN domain or the SD domain were generated (8, 9). Characterization of mice lacking the TN domain (*Nkx2.2^TNMut^*), resulted in dysfunctional β cells that were either immature or transdifferentiated into α cells. Molecular experiments showed the NKX2.2 TN domain is required for the repressive activity of NKX2.2 by recruiting the Transducer-like enhancer of split 3 (TLE3)/Groucho-related gene 3 (GRG3) and Histone deacetylase 1 (HDAC1) to many repressed targets, including the master α cell regulator Aristaless homeobox gene *(Arx)* promoter (8). This study showed the importance of the TN domain as a major contributor to *Nkx2.2*’s role of repressing non-β cell genes in β cells.

Characterization of mice lacking the SD domain (*Nkx2.2^SDMut^*) resulted in elevated blood glucose levels due to down regulation of β cell maturation and identity genes, among other phenotypes (9). It is still unknown what cofactor proteins the NKX2.2 SD domain interacts with to allow this activation role. These previous studies showed that NKX2.2 activates β cell genes and represses non-β cell genes; however, the mechanisms underlying these contrasting roles of NKX2.2 in the same cell type is currently unknown. To better characterize *Nkx2.2’s* function in β cells it is necessary to identify the cofactor proteins NKX2.2 interacts with as well as the roles of those proteins themselves in the β cell.

In this study, we set out to uncover essential cofactor proteins of NKX2.2 and determine their role in β cells. We identified Chromodomain Helicase DNA-binding Protein 4 (CHD4) as a NKX2.2 interacting factor and generated mice that deleted *Chd4* specifically in the developing β cells. These studies demonstrated that CHD4 is important for the proper maturation and function of β cells and the structural integrity of islets. Furthermore, we uncovered both NKX2.2 dependent and independent roles for CHD4 during β cell maturation.

## Results

### CHD4 and NuRD complex proteins interact with NKX2.2 in pancreatic β cells

**Fig. 1.**
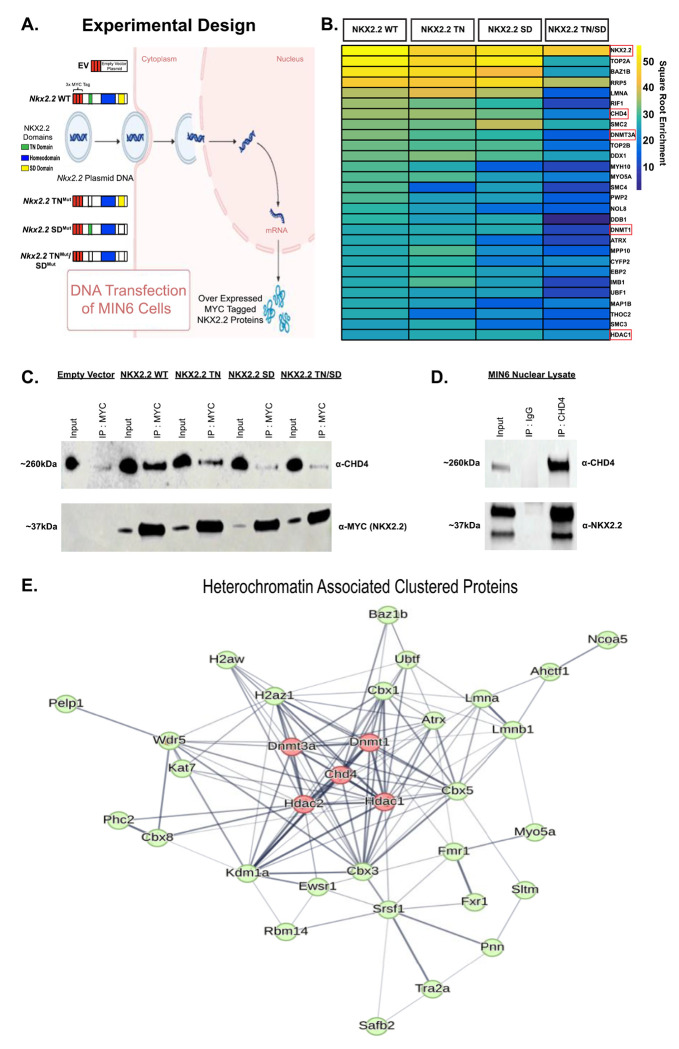
NKX2.2 interacts with chromatin modifying protein CHD4. (A) Schematic for transfection of *Nkx2.2* plasmids with WT and mutant domains into MIN6 β cell culture line for IP-MS. Empty Vector (EV) used as control. (B) Heatmap showing a subset of the highest enriched proteins to come out of the NKX2.2 IP-MS for WT NKX2.2 and each domain mutant. Color represents square root normalization of enrichment. (C) Western blot of MYC tag IP in MIN6 nuclear lysate. (D) Western blot of CHD4 IP in MIN6 nuclear lysate. (E) Heterochromatin group cluster from string database clustering (18) showing overlapping CHD4 and NKX2.2 interacting proteins.

NKX2.2 has been shown to function as a repressor and activator in pancreatic islets (5, 7–9). To identify cofactors of NKX2.2 that may influence its function in pancreatic β cells, we performed an unbiased screen for interacting proteins. Mouse Insulinoma 6 (MIN6) β cells were transfected with *Nkx2.2*-encoding plasmids that incorporated 3 myc epitope tags at the N-terminus of the full-length NKX2.2 protein and several previously characterized *Nkx2.2* mutant derivatives (TN^mut^, SD^mut^, TN^mut^/SD^mut^) (8, 9) (Figure 1A). A myc-tagged empty vector (EV) was used as a control. This strategy not only allowed an unbiased identification of NKX2.2 cofactors but also potentially identified cofactors that were dependent on specific NKX2.2 protein domains. An anti-MYC antibody was used to immunoprecipitate (IP) the NKX2.2 proteins, followed by mass spectrometry (MS) analysis. The IP-MS with NKX2.2 wild type (WT) identified the most interacting proteins with 211 proteins enriched at least 2-fold in abundance compared to the EV control. From the NKX2.2 TN^mut^ and NKX2.2 SD^mut^ pulldowns, we identified 169 and 143 interacting proteins, respectively, that were enriched at least 2-fold in abundance compared to the EV control. Interestingly, from the NKX2.2 TN^mut^/SD^mut^ pulldown, we only identified approximately 84 interacting proteins, suggesting that deletion of the SD domain disrupted a substantial number of interactions, independently and in cooperation with the TN domain.

The IP-MS identified DNMT3A, DNMT1 and HDAC1, proteins that had been previously shown to interact with the NKX2.2 TN domain to repress the *Arx* locus in β cells (8, 10). In addition to identifying known interacting proteins, several members of the Nucleosome Remodeling and Deacetylase (NuRD) complex, including the functional enzymatic protein Chromodomain Helicase DNA-binding Protein 4 (CHD4) were identified in the screen (Figure 1B). CHD4 showed the highest enrichment in the NKX2.2 WT and the NKX2.2 TN^mut^ samples and the interaction appeared to be weakened in the NKX2.2 SD^mut^ and NKX2.2 TN^mut^/SD^mut^ samples. The interaction between the NKX2.2 domains and CHD4 was validated through Co-Immunoprecipitation (Co-IP) followed by Western blot (Figure 1C), which showed a similar pattern of enrichment observed in the IP-MS. The interaction between NKX2.2 and CHD4 was disrupted to a greater extent in the NKX2.2 SD^mut^ and NKX2.2 TN^mut^/SD^mut^ samples, suggesting the SD region may be the main domain facilitating the interaction of NKX2.2 and CHD4.

Both the NuRD complex and CHD4 specifically have been implicated as necessary for proper development, maturation and function of many different tissues and cells, often acting as gene repressors by deacetylating histones and remodeling chromatin (11–15), although activation of genes has been noted as well (16, 17). To further validate an interaction between CHD4 and NKX2.2, we immunoprecipitated CHD4 from MIN6 nuclear lysate followed by MS. As expected, this identified the other NuRD complex components (HDAC1 and 2, MTA1, 2 and 3, RBBP 4 and 7, MBD 2 and 3), many of which were also present in the NKX2.2 WT MS. NKX2.2 was also identified in the CHD4 IP-MS. We further verified the interaction between CHD4 and NKX2.2 by pulling down CHD4 in MIN6 nuclear lysate and western blotting for endogenous NKX2.2 (Figure 1D).

Comparison between proteins that interacted with either NKX2.2 WT and CHD4 identified 112 proteins as cofactors of both NKX2.2 and CHD4. Greater than half of the significantly enriched protein cofactors identified in the NKX2.2 WT MS were also significant CHD4 protein cofactors and a hypergeometric test confirmed that this represents a statistically significant enrichment (p < 0.001). A String database (18) was used to group the 112 proteins using K means clustering into 3 main clusters (Supplementary Figure 1A). CHD4 and 32 other proteins were clustered as a protein group associated with heterochromatin. Interestingly, in addition to HDAC1 and HDAC2, DNMT3A and DNMT1 interacted with both CHD4 and NKX2.2 (Figure 1E). Due to the known repressor functions of DNA methyltransferases and histone deacetylases (19), we hypothesized that NKX2.2 recruits CHD4 to specific sites in the β cell genome to facilitate repression by closing chromatin and repressing non-β cell genes. To test this hypothesis, we investigated the role of CHD4 in mouse β cell development, maturation and function.

### Pancreas knock out of Chd4 leads to blood glucose and body weight defects

*Chd4* is expressed in all pancreatic islet cells types (20) and has a role in regulating adult β cell function through an interaction with Pancreas Duodenum Homeobox1 (PDX1) (21, 22); however, its role in regulating islet development and maturation, where NKX2.2 plays a major role, had not yet been assessed. To determine whether CHD4 functions during pancreas development we generated mice carrying a *Chd4* floxed (*Chd4^(fl/fl)^)* (16) allele and a *Pdx1^(Cre/+)^* (23) allele which would remove CHD4 from the entire pancreas and duodenum at the onset of pancreas formation (Supplementary Figure 1B). The *Chd4^(fl/fl)^; Pdx1^(Cre/+)^* mice displayed a significant increase in the *ab libitum* blood glucose as early as postnatal day 2 (P2) and through 4 weeks of age compared to *Chd4^(fl/fl)^* and *Chd4^(fl/+)^; Pdx1^(Cre/+)^* control mice (Supplementary Figure 1C). Morphometric analysis using immunofluorescent (IF) staining did not reveal an alteration of islet cell numbers or islet architecture at P2; but there was a severe disruption in islet structure at 4 weeks of age (Supplementary Figure 1D). However, there was also a significant decrease in body weight of mutant *Chd4^(fl/fl)^; Pdx1^(Cre/+)^* mice compared to *Chd4^(fl/fl)^* and *Chd4^(fl/+)^; Pdx1^(Cre/+)^* mice at P2 and 4 weeks of age (Supplementary Figure 1C). This suggests that removing *Chd4* from the pancreatic exocrine, ductal, and intestinal duodenal cells, in addition to loss of *Chd4* from the pancreatic endocrine lineage may disrupt the proper secretion of digestive enzymes and/or digestion of foods. Since assessment of endocrine pancreas function could be confounded by the defects in these other tissues that caused significant body weight loss, we chose to focus on the role of CHD4 specifically in the developing β cells.

### Chd4 βKO causes no overt developmental phenotype

**Fig. 2.**
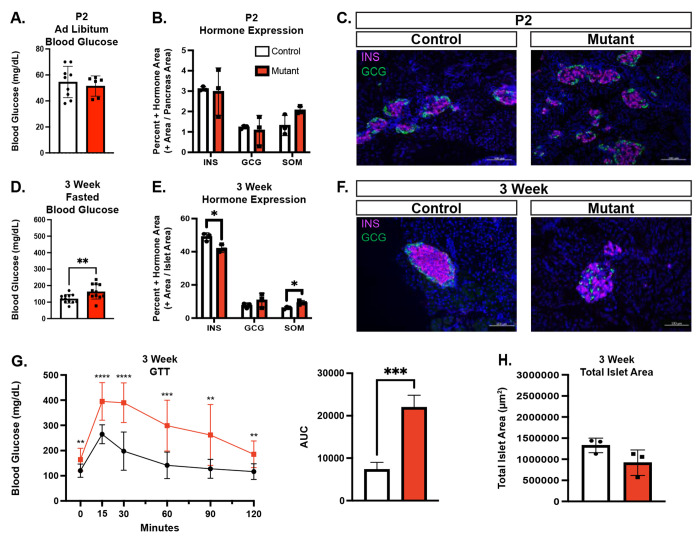
*Chd4* βKO mice are hyperglycemic with disrupted islet architecture and dysfunctional β cells. Unless otherwise noted, mouse data consists of control (*Chd4^fl/fl^* : white bars) and mutant/*Chd4* βKO (*Chd4^fl/fl^;* *Ins1^Cre/+^*: red bars) genotypes. (A) Measurements of *Ad libitum* blood glucose for P2 control (n =6) and mutant (n=9) mice. (B) Islet hormone quantification at P2 for Insulin (INS), Glucagon (GCG) and Somatostatin (SOM) for control (n=3) and mutant (n=3) mice. (C) Representative immunofluorescent images for P2 control and mutant mice showing DAPI (blue), Insulin (INS, Magenta), and Glucagon (GCG, green). Scale bars: 100 μm. (D) Measurements of fasted blood glucose for 3-week control (n =12) and mutant (n=12*)* mice. (E) Islet hormone quantification at 3-weeks for Insulin (INS), Glucagon (GCG) and Somatostatin (SOM) for Control (n=3) and Mutant (n=3) mice. (F) Representative immunofluorescent images for 3-week control and mutant mice showing DAPI (blue), Insulin (INS, Magenta), and Glucagon (GCG, green). Scale bars: 100 μm. (G) Glucose Tolerance Test (GTT) of 3-week control (black line, n=12) and mutant (red line, n=12) mice (multiple comparison two-tailed Student’s *t*-test). Area under the curve (AUC) measurement for GTT. All points normalized to 0 minutes time point prior to AUC calculation. (H) Quantification of 3-week control (n=3) and mutant (n=3) total islet area. For all figures, *P≤0.05, **P≤0.01, ***P≤0.001, ****P≤0.0001; two-tailed Student’s *t*-test unless otherwise specified.

To characterize the function of CHD4 in developing β cells from the onset of their differentiation, we generated *Chd4^(fl/fl)^; Ins1^(Cre/+)^* (24) mice (hereafter referred to as *Chd4* βKO mice). Deletion of the CHD4 protein specifically from the β cell lineage was confirmed through Western blot analysis using mouse islet lysate (Supplementary Figure 2A). Because CHD4 is present in other islet cell types, we further validated the β cell deletion using IF staining (Supplementary Figure 2B). At all ages tested, deletion of *Chd4* from the β cells did not result in a body weight phenotype (Supplemental Figure 2C). To characterize *Chd4* βKO mice, we performed morphometric analysis and assessed blood glucose at postnatal day 2 (P2). Unexpectedly, given the severe developmental phenotypes associated with deletion of *Pdx1* or *Nkx2.2* in developing β cells (4, 5, 25–27), and the known interaction of PDX1 and NKX2.2 with CHD4, the neonatal *Chd4* βKO mice had normal blood glucose levels (Figure 2A), as well as no change in the hormone area of insulin, glucagon or somatostatin (Figure 2B), compared to control mice. There were also no apparent alterations in the islet architecture or structure when comparing *Chd4* βKO mouse islets to control mouse islets (Figure 2C), suggesting that CHD4 is not necessary for specification or early development of the β cell lineage.

### Chd4 βKO mice are glucose intolerant by 3 weeks of age

At 3 weeks of age, shortly after weaning, combined male and female *Chd4* βKO mice had significantly higher fasting blood glucose levels (Figure 2D) when compared to control mice that included *Chd4^(fl/fl)^, Ins1^(Cre/+)^* (*Cre* only control) and *Chd4^(fl/+)^; Ins1^(Cre/+)^* (Het) mice. Because none of the control mice of any genotype displayed defects in fasting blood glucose levels or glucose tolerance (Supplementary Figure 3), subsequent experiments included only *Chd4^(fl/fl)^* (control) and *Chd4^(fl/fl)^*; *Ins1^(Cre/+)^* (*Chd4* βKO) mice. When assessing sex separately at 3 weeks, only female *Chd4* βKO mice showed significantly higher fasting and *ad libitum* blood glucose levels compared to controls and were glucose intolerant (Supplementary Figure 4A). Morphometric analysis performed on combined male and female mice at 3 weeks of age showed a significant decrease in the insulin positive hormone area, no change in the glucagon positive area, and a slight but significant increase of the somatostatin positive hormone area in *Chd4* βKO compared to control mice (Figure 2E). In addition to the changes in hormone expression area, islet architecture was disrupted in *Chd4* βKO mice compared to control mice; glucagon and somatostatin expressing cells, typically present at the periphery of the islet, were present in the islet core where only β cells are normally seen (Figure 2F). Interestingly, we did not observe the formation of polyhormonal cells, as was seen in the Nkx2.2 βKO mice (7). Lastly, combined male and female *Chd4* βKO mice were significantly more glucose intolerant (Figure 2G) when compared to control mice. Interestingly, the significant decrease in the insulin positive hormone area at 3-weeks was not accompanied by a change in the total islet area between *Chd4* βKO and control mice (Figure 2H).

### Chd4 βKO mice phenotype worsens through 6 weeks of age and are diabetic by 10 weeks of age

**Fig. 3.**
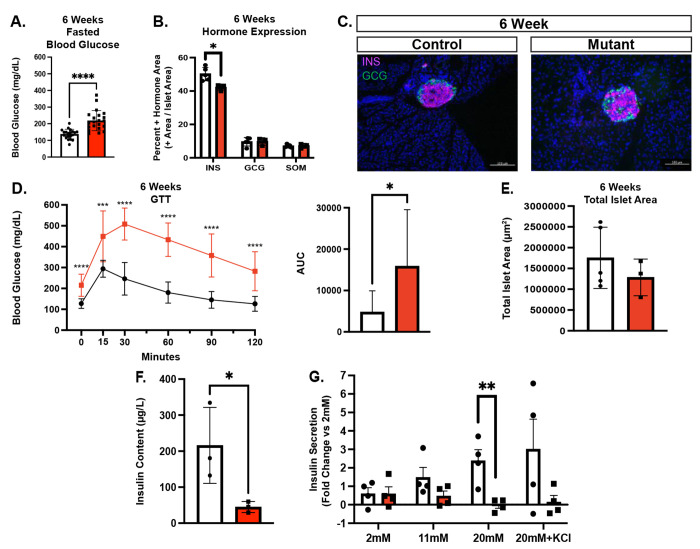
*Chd4* βKO mice are hyperglycemic with disrupted islet architecture and dysfunctional β cells. Unless otherwise noted, mouse data consists of control (*Chd4^fl/fl^* : white bars) and mutant/*Chd4* βKO (*Chd4^fl/fl^;* *Ins1^Cre/+^*: red bars) genotypes. (A) Measurements of fasted blood glucose for 6-week control (n =20) and mutant (n=23*)* mice. (B) Islet hormone quantification at 6-weeks for Insulin (INS), Glucagon (GCG) and Somatostatin (SOM) for control (n=5) and mutant (n=3) mice. (C) Representative immunofluorescent images for 6-week control and mutant mice showing DAPI (blue), Insulin (INS, Magenta), and Glucagon (GCG, green). Scale bars: 100 μm. (D) Glucose Tolerance Test (GTT) of 6-week control (black line, n=13) and mutant (red line, n=13) mice (multiple two-tailed Student’s *t*-test). Area under the curve (AUC) measurement for GTT. All points normalized to 0 minutes time point prior to AUC calculation. (E) Quantification of 6-week control (n=5) and mutant (n=3) total islet area. (F) Insulin content from dissected mouse pancreas of 6-week Control (n=3) and Mutant (n=3). (G) Glucose Stimulated Insulin Secretion (GSIS) assay of 6-week control and mutant pancreas slices. Conditions were 2mM glucose, control (n=4) and mutant (n=4), 11mM glucose, Control (n=4) and Mutant (n=4), 20mM glucose, control (n=4) and mutant (n=4), 20mM glucose + KCl, control (n=4) and mutant (n=4). Fold Change is each condition normalized to 2mM basal secretion levels. For all figures, *P≤0.05, **P≤0.01, ***P≤0.001, ****P≤0.0001; two-tailed Student’s *t*-test unless otherwise specified.

By 6 weeks of age, fasting and *ad libitum* blood glucose levels in both the male and female *Chd4* βKO mice were significantly elevated compared to controls (Figure 3A, Supplementary Figure 4B). There was still a significant decrease in the insulin positive hormone area between *Chd4* βKO mice and control mice, while both glucagon and somatostatin positive hormone areas were unchanged (Figure 3B). At this age, islet architecture continued to be disrupted with peripheral glucagon and somatostatin expressing cells present in the interior of the islets, but there was still no evidence of polyhormonal cells (Figure 3C). Also at 6 weeks, the *Chd4* βKO mice became progressively more glucose intolerant (Figure 3D), though the significant decrease in the insulin positive hormone area at 6 weeks was still not accompanied by a change in the total islet area between *Chd4* βKO and control mice (Figure 3E), suggesting β cells were maintained, but not expressing insulin.

By 10 weeks of age, the *Chd4* βKO male mice became severely diabetic compared to controls, displaying significantly lower body weight, significantly higher fasted and *ad libitum* blood glucose levels, severe glucose intolerance (Supplementary Figure 4C) and dehydration due to excessive urination. The hyperglycemic phenotype and health of the female mice also continued to worsen through 10 weeks of age (Supplementary Figure 4C), although the decline progressed more slowly, and female mice did not become as severely diabetic until approximately 15 weeks of age (data not shown). Overall, the *Chd4* βKO mice displayed a significant hyperglycemia phenotype with significantly reduced insulin expression and glucose intolerance starting as early as 3 weeks of age. *Chd4* βKO mice progressed to diabetes by 10-15 weeks of age. Further phenotypic and molecular analysis focused on the 3-week - 6-week timepoints to minimize secondary affects associated with the diabetic phenotype.

### Chd4 βKO mice have reduced insulin content and defective insulin secretion

In our attempts to isolate islets to assess the functionality of *Chd4 β*KO β cells we identified a striking islet fragility phenotype; the isolated *Chd4 β*KO mutant islets rapidly dissociated upon manipulation. To initially circumvent this challenge, insulin content from intact whole pancreata isolated from 6-week-old mice was measured. *Chd4* βKO mice displayed significantly less insulin content compared to control mice (Figure 3F). To assess insulin secretion, live pancreas slices (28) were generated, and used to perform Glucose Stimulated Insulin Secretion (GSIS) assays. These experiments demonstrated that *Chd4* βKO islets had normal basal insulin secretion at 2mM glucose, trending lower insulin secretion at 11mM glucose, significantly lower insulin secretion at 20mM glucose and trending lower insulin secretion at 20mM glucose + KCl conditions compared to controls (Figure 3G). Taken together, these studies suggested that the *Chd4* βKO islets had both reduced insulin production and an impaired glucose response.

### Chd4 mutant β cells show immature transcriptional landscape

**Fig. 4.**
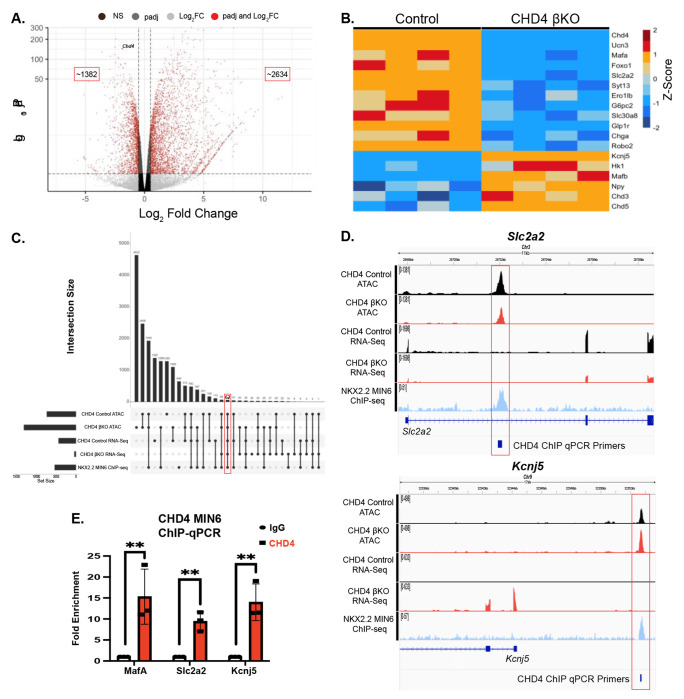
CHD4 binds and regulates essential pancreatic β cell genes. (A) Volcano plot showing the distribution of differentially expressed genes (DEGs) in *Chd4* βKO mice. (B) Heatmap showing a subset of differentially expressed β cell genes between control and *Chd4* βKO mice. Heat map is mean centered using z-score scale. (C) UpSet plot showing all the genes that overlap in various combinations throughout the five datasets, *Chd4* control and βKO ATAC-seq, *Chd4* and *Nkx2.2* βKO RNA-seq and *Nkx2.2* ChIP-seq datasets. 62 genes overlapped in all five datasets. (D) IGV tracks showing genome locations and sequencing read peaks of *Chd4* control and βKO ATAC-seq, *Chd4* control and βKO RNA-seq and *Nkx2.2* ChIP-seq datasets overlapped with CHD4 binding sites through qPCR primer locations for *Slc2a2* and *Kcnj5*. (E) Quantification of qPCR using fold enrichment over IgG of *MafA, Slc2a2* and *Kcnj5* representing binding sites of CHD4 at these genes (two-tailed Student’s *t*-test). **P≤0.01.

Because CHD4 is a chromatin remodeling factor that interacts with NKX2.2, a transcription factor, it was important to identify the transcriptional defects that could explain the observed β cell dysfunction. To purify the β cell population we introduced an *Ins2^(GFP/+)^* allele (29) into the control and *Chd4* βKO mice for Fluorescence Activated Cell Sorting (FACS) (Supplementary Figure 5). Transcriptome analysis on β cells isolated from 4-week-old mice revealed a large number of genes significantly upregulated and downregulated in the *Chd4* βKO mice vs. controls (Figure 4A). Consistent with efficient conditional deletion of the *Chd4* locus, *Chd4* was one of the most significantly downregulated genes. In addition, there was a significant decrease in many essential β cell genes/factors including *Ucn3, MafA* and *Foxo1*. There was also a reduction in many of the genes involved in glucose sensing and insulin secretion including *Slc2a2* (GLUT2), *Syt13, Ero1lβ, G6pc2*, *Slc30a8* (ZnT8), and *Glp1r* (Figure 4B). Interestingly, *Chd4* βKO mice also displayed a significant increase in genes normally expressed in immature β cells including *MafB* and *Npy* or pancreas disallowed genes such as *Hk1*. Consistent with the lack of polyhormonal cells in the IF analysis, there was not a corresponding increase in non-β endocrine cell genes. Lastly, we also observed significant downregulation of *Robo2* (Figure 4B). Previous studies demonstrated that deletion of *Robo1* and *Robo2* from β cells (*Robo* βKO) caused islets to easily dissociate (30, 31). Deletion of *Robo2* alone from the β cells was sufficient to disrupt the islet architecture (32, 33), similar to the phenotypes observed in the *Chd4* βKO mice. Overall, transcriptional analysis of the *Chd4* βKO β cells identified disruption of many β cell maturity and functional markers suggesting this was the underlying cause of the severe CHD4 mutant phenotype.

### CHD4 affects NKX2.2 - associated β cell gene regulation through chromatin remodeling

After identifying CHD4 as an NKX2.2 co-factor, we were specifically interested in β cell genes that were co-regulated by NKX2.2 and CHD4 and were associated with changes in the chromatin landscape when CHD4 was deleted. To detect changes in chromatin landscape, we reanalyzed a previously published CHD4 Control ATAC-seq vs. adult CHD4 βKO ATAC-seq dataset (22). As expected for a chromatin modifier protein, there were greater than 52,500 total peaks of significantly altered chromatin (padj < 0.05) when CHD4 was disrupted. The majority of peaks (39,956) displayed regions of more open chromatin, although there were a number of regions (12,843) that became more closed. To identify genes that are NKX2.2-dependent direct targets of CHD4, we identified genes that were bound by NKX2.2, dysregulated in both the *Nkx2.2* βKO and *Chd4* βKO islets and displayed changes in chromatin accessibility. 62 genes were present in all five datasets (Figure 4C), including *Ucn3, Slc2a2, G6pc2, Slc30a8, Kcnj5 and Npy*, suggesting that these genes represent potential direct targets of CHD4 and NKX2.2.

### β cell maturation genes are direct targets of NKX2.2 and CHD4

To validate the subset of potential NKX2.2-dependent direct gene targets of CHD4 that correlated with the observed phenotypes, we performed chromatin precipitation followed by qPCR (ChIP-qPCR) in MIN6 cells on genes that either were deactivated or activated in the absence of CHD4 and NKX2.2. We included the previously characterized CHD4 bound *MafA* regulatory region 3 as a positive control for these experiments (21, 34). *Slc2a2*, the gene encoding the β cell glucose transporter GLUT2 is bound by NKX2.2, displayed reduced chromatin accessibility in the CHD4 βKO ATAC samples and was significantly down regulated when either *Nkx2.2* or *Chd4* were deleted from β cells (Figure 4D). Alternatively, *Kcnj5* (GIRK4), a channel that is normally not expressed in pancreatic β cells and responds to somatostatin and epinephrine signaling, is also bound by NKX2.2, had increased chromatin accessibility in the CHD4 βKO samples and was up regulated when either *Nkx2.2* or *Chd4* were deleted from β cells (Figure 4D). CHD4 ChIP-qPCR validated both genes were bound by CHD4 demonstrating they were direct targets that are co-regulated by NKX2.2 and CHD4 (Figure 4D, 4E). Closer analysis in a subset of genomic regions identified in these datasets, indicates that NKX2.2 binds to and facilitates the recruitment of CHD4 to alter the chromatin landscape to allow appropriate gene transcription.

### Chd4 mutant β cells show disrupted calcium signaling

**Fig. 5.**
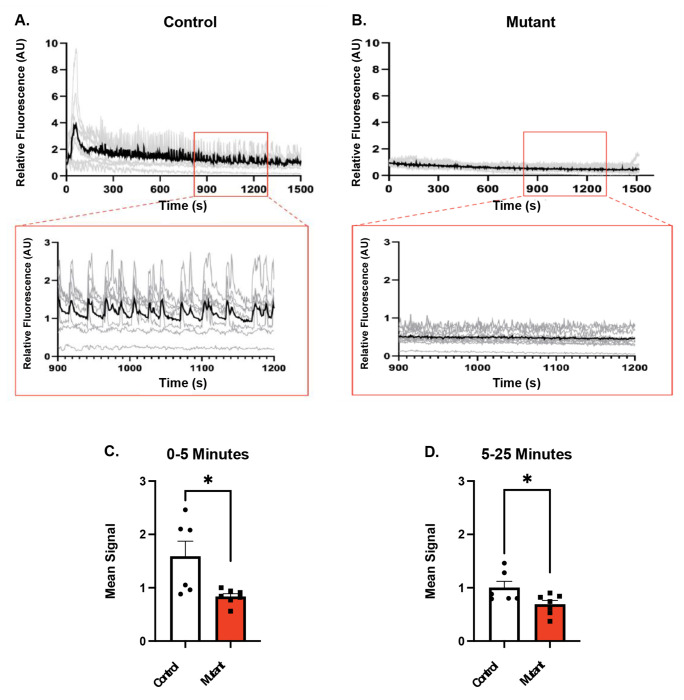
Calcium signaling is disrupted in Chd4 βKO pancreas slice culture. (A) Time-course of the Fluo4 Ca^2+^ sensor fluorescence for one islet from a control pancreas slice following elevation of glucose to 11mM. Each gray trace is one cell from the islet, and the black trace is the average fluorescence of all cells from the islet. Fluorescence is normalized to the mean fluorescence at 2mM glucose. Inset box is zoomed in view from 900 to 1200 seconds showing coordinated calcium oscillations in the control islet. (B) Time-course of the Fluo4 Ca^2+^ sensor fluorescence for one islet from mutant pancreas slice, as in A. (C) Quantification of mean Fluo4 signal for the first 5 minutes after Ca^2+^ elevation, for control (n=6) and mutant (n=6) pancreas slices. Mean signal = average fluorescence over indicated time (two-tailed Student’s *t*-test). (D) Quantification of mean signal for 5 minutes to 25 minutes after Ca^2+^ elevation for control (n=6) and mutant (n=6) pancreas slices, as in C (two-tailed Student’s *t*-test). *P≤0.05.

With the observed down regulation of *Robo2 in the Chd4* βKO islets, and previous research showing the deletion of *Robo1* and *Robo2* from β cells causes both fragile islets and disrupts calcium signaling (30), we next tested whether disrupted calcium signaling contributed to the insulin secretion defects. To circumvent the islet fragility phenotype, we tested calcium signaling in *Chd4* βKO islets using the live pancreas slice preparation (28). Using the Ca^2+^ sensor, Fluo-4, we measured the Ca^2+^ response in control and *Chd4* βKO pancreatic slices upon elevated glucose. Following addition of 11mM glucose, islets within control slices showed an initial elevation in Ca^2+^, followed by synchronized pulsatile waves of Ca^2+^ (Figure 5A, Supplementary Video 1), which is similar to the patterns seen in control isolated islets. However, the islets within *Chd4* βKO slices lacked any Ca^2+^ response to elevated glucose, including lack of an initial elevation in Ca^2+^, and lack of pulsatile waves of Ca^2+^ (Figure 5B, Supplementary Video 2). The mean Ca^2+^ elevation, expressed as the mean fluorescence increase from 0-5 minutes and 5-25 minutes each had significantly lower signal in the *Chd4* βKO slices than in the control slices (Figure 5C, 5D). Therefore, the *Chd4* βKO slices show a substantial decline in the Ca^2+^ response to glucose, which could cause the impaired glucose-stimulated insulin secretion and the overall dysfunction of the *Chd4* βKO mutant β cells.

## Discussion

β cell maturation is necessary to achieve proper glucose-responsive β cells and when disrupted can cause dysfunction and loss of identity in β cells leading to hyperglycemia. Prior studies in our lab have shown the essential role of NKX2.2 in the proper development, maturation and function of pancreatic β cells. Given its diverse functions, NKX2.2 likely interacts with co-factors to exert its activities. In this study, we identified about 200 proteins that potentially interact with NKX2.2 to facilitate transcriptional regulation in the β cell. Many of the interactions were mediated by either the TN domain, SD domain or both. The IP-MS successfully validated previously characterized interactions between NKX2.2 and HDAC1, DNMT3A, and DNMT1 that were facilitated through the NKX2.2 TN domain and function to repress non-β cell genes in β cells (8). In addition, we identified novel interactions between NKX2.2 and several members of the Nucleosome Remodeling and Deacetylase (NuRD) complex, including CHD4, an enzymatic component of the complex. We initially theorized that the interaction between NKX2.2 and CHD4 would facilitate transcriptional repressor activities that were mediated by the NKX2.2 TN domain. However, the MS and Co-IP experiments suggested that the interaction with CHD4 is predominantly mediated by the NKX2.2 SD domain. Deletion of *Chd4* from β cells affected both the repression and activation of essential genes, leading to the disruption of the proper maturation and function of β cells.

Although *Chd4* was deleted from β cells at the onset of insulin expression at approximately gestational embryonic stage e12.0, the *Chd4* βKO mice did not display an overt phenotype at P2, suggesting the lack of an overt developmental phenotype. This result was somewhat surprising given its interaction with NKX2.2, which is essential for proper β cell development (4, 5). It is possible that *Chd4* is dispensable for embryonic β cell development. Alternatively, although we confirmed that CHD4 was efficiently removed from the majority of the adult β cells (Supplementary Figure 2B), a small proportion of β cells retained CHD4 expression and these remaining β cells could be sufficient to sustain glucose regulation through the early postnatal stages. Alternatively, CHD4 function may also be compensated by other family members including CHD3 and CHD5, which were both upregulated in the *Chd4* βKO (Figure 4B). However, if CHD3 and CHD5 do compensate for CHD4 during the developmental window, they appear to be unable to fully do so in β cell maturation and function postnatally.

Deletion of *Chd4* from the developing β cell lineage had a substantial impact on the maturation and function of β cells. *Chd4* βKO mice were glucose intolerant and hyperglycemic by 3 weeks of age with their symptoms worsening with age. Interestingly, a recent study that used a *MIP:Cre^ERT^* to remove *Chd4* from fully mature adult β cells (22) caused impaired insulin secretion; however, the phenotype was less severe than when *Chd4* was deleted from developing β cells, suggesting that in β cells CHD4 may play a stronger role in initiating chromatin modifications to establish a transcriptional program than maintaining chromatin structure. In addition, several important β cell transcription factors, including *Foxo1*, and β cell maturation factors such as *Ucn3* were not affected when *Chd4* was deleted from adult β cells, suggesting that maintenance of β cell identity and maturation is not dependent on CHD4. However, *Robo2* and *Slc2a2* gene transcription was disrupted in the *Chd4^fl/fl^; MIP:Cre^ERT^* mice, suggesting CHD4 is required to maintain β cell integrity and functional status.

This study begins to parse out novel roles of CHD4 in establishing the correct chromatin landscape and transcriptional status during the formation and maturation of β cells vs. its role in maintaining β cell functions. Davidson et al., (21) identified an interaction between CHD4 and PDX1 and showed the interaction of CHD4 facilitated by PDX1 may also be tuned through glucose sensing in the β cell. Though we have not determined if NKX2.2 and CHD4’s interaction is facilitated through glucose sensing, the NKX2.2 MS did identify PDX1 as a potential NKX2.2 interacting factor. Consistently, NKX2.2 and PDX1 co-regulate many essential regulatory sites in the β cell genome including at *Mafa, Slc2a2 and Robo2*, although not all sites are shared, such as at *Kcnj5* (7, 35). This leads us to believe that some actions of CHD4 in the β cells are facilitated through its recruitment from both NKX2.2 and PDX1. Further work is needed to determine when CHD4 is recruited solely by NKX2.2, PDX1 or both and how this differential recruitment affects its molecular functions.

At the end of the pancreatic β cell maturation window, at approximately 3 weeks of age, the *Chd4* βKO mice were glucose intolerant with elevated blood glucose levels and a reduction in the insulin area. Since there was not a substantial change in total islet area in these mutant mice (Figure 2H, 3E), the observed reduction in insulin positive area was most likely caused by a reduction in insulin production rather than a decrease in β cell numbers. Consistently, we did not observe changes in Ki-67 staining for proliferation or TUNEL staining for cell death between *Chd4* βKO and control mice (Supplementary Figure 6A, 6B). We were unable to perform lineage tracing experiments to fully characterize whether β cells were still present but not producing insulin because the *R26R:tdTomato* locus is closely linked to the *Chd4* locus on mouse chromosome 6 precluding our ability to generate a *Chd4^(fl/fl)^; Ins1^(Cre/+)^; Rosa^(Tomato/Tomato)^* lineage tracing mouse.

The identification of *Robo2* as significantly down regulated in the absence of Chd4, suggests that loss of *Robo2* may contribute to the fragile islet phenotype as well as the disrupted islet architecture and impaired calcium signaling (30, 32, 33). However, deletion of *Robo2* alone from β cells did not cause disruption of maturation and β cell identity markers, which is seen in our study. This suggests the loss of β cell maturation and identity may be driven by direct targets of CHD4 such as *MafA* and *Slc2a2*. Furthermore, in addition to *Robo2*, multiple genes involved in cell-to-cell and cell-to-matrix junctions were also significantly down-regulated including many genes involved in desmosomes (*Jup, Pkp2, Dsc2*) which have been shown to affect tissue architecture and structure (36–38), suggesting additional CHD4 targets may contribute to the fragile islet phenotype. Lastly, the upregulation of *Kcnj5* could contribute to the disrupted calcium signaling as well. *Kcnj5* encodes the GIRK4 channel, which is not normally expressed in β cells but can respond to somatostatin and epinephrine signaling. These signals can potentially hyperpolarize the β cell, affecting calcium signaling and insulin secretion (39).

In other tissue and cell types, CHD4 has been shown to either activate genes, including those associated with lineage programs (16, 17), or repress genes associated with alternative lineage programs (14, 40). We have shown that in pancreatic β cells, CHD4 has the ability to bind at and either activate or repress transcription of β cell genes. NKX2.2 facilitates the ability of CHD4 to bind at and manipulate the chromatin state at certain genes, allowing CHD4 to activate essential maturation and functional β cell genes, such as *Slc2a2* while at the same time repressing genes that will be detrimental to β cell function, such as *Kcnj5*. However, we did observe nearly 1000 more genes up regulated overall with the deletion of *Chd4* as well as almost three times the number of regions that gained open chromatin in the CHD4 βKO ATAC-seq. This suggests that although CHD4 can both activate and repress genes in β cells, CHD4 is still primarily acting as a repressor. Additional studies would be necessary to identify whether the distinct NKX2.2 domains confer either NKX2.2 and/or CHD4 combined roles as repressors or activators in β cells. It is possible that HDAC1 and the NuRD complex are recruited by the NKX2.2 TN domain to repress certain β cell genes, whereas activation of a different subset of β cell genes via CHD4 is facilitated by the NKX2.2 SD domain.

Overall, understanding the transcriptional network that facilitates the proper development, maturation and function of pancreatic β cells can help lead to better treatment options for diseases like diabetes. This study increases our understanding of how essential transcription factors in the β cell, like NKX2.2, maintain a proper transcriptional state through the interaction with cofactor proteins such as CHD4 to allow the proper maturation and function of β cells.

## Materials and Methods

### Cell Line and Cell Culture

Mouse Insulinoma (MIN6) β cell culture line was originally created by the Yamamura lab (41) and cultured at 37°C and 5% CO_2_. MIN6 cells were passaged every 7 days and fed media containing Dulbecco’s Modified Eagle Medium (DMEM) high glucose, with L-glutamine, without Sodium pyruvate and HEPES (Gibco, Cat. # 11965-092) and supplemented with 10% FBS (Gibco, Cat. #A5669701), 5% PenStrep (10,000U/mL Penicillin-Streptomycin, Gibco, Cat. #15140-122) and .00040% βME.

### Nkx2.2 Domain Variant Plasmid Transfections

MIN6 cells were forward transfected with 31.5 μg of one of the following *Nkx2.2* plasmids: pcDNA3-3x Myc-EV, pcDNA3-3x Myc-Nkx2.2 WT, pcDNA3-3x Myc-Nkx2.2 TN^mut^, pcDNA3-3x Myc-Nkx2.2 SD^mut^, or pcDNA3-3x Myc-Nkx2.2 SD^mut^ / TN^mut^. Lipofectamine-3000 was used as the transfection reagent (Invitrogen, Cat. #L3000015) and MIN6 cells were transfected for 72 hours prior to protein extraction using the Nuclear Extract Kit (Active Motif, Cat. #40010). Protein concentration was assessed using the DC assay kit (Bio-Rad, Cat. #5000112).

### Co-Immunoprecipitation for Mass Spectrometry

For mass spectrometry (mass spec) of transfected Myc tagged NKX2.2 proteins, pull downs were performed by incubating Anti-c-MYC magnetic beads (Pierce, Cat. #88843) with samples for 1 hour with mixing at 4°C. Samples were then washed with a Co-IP Buffer (50mM HEPES, 1mM EDTA, 5% glycerol, 0.5% Triton-X, 500nM NaCl). For mass spec of endogenous CHD4 protein, pulldowns were performed in WT MIN6 cells using the Nuclear Complex Co-IP Kit (Active Motif, Cat. #54001) per the manufacturer’s instructions. Briefly, 500 μg of WT MIN6 nuclear extract was incubated in IP High Buffer (no detergent and no NaCl) using 5 μg of CHD4 rabbit antibody (D8B12, CST, Cat. #11912) or 5 μg of a control IgG rabbit antibody (CST, Cat. #2729), overnight with mixing. The second day, samples were incubated with Anti-Protein-A magnetic beads (Pierce, Cat. #88846) for 1 hour with mixing at 4°C. Samples were then washed using the IP High Buffer (no detergent and no NaCl) with and without BSA.

For both the NKX2.2 and CHD4 samples, 4x Laemmli buffer (Bio-rad, Cat. #1610747) plus βME was used as a sample buffer and samples were boiled for 5 minutes at 95°C and loaded in a Bio-Rad Mini-PROTEAN TGX Gel (4-20%, 10-well, 50 μL, Bio-rad, Cat. # 456-1094). Once all samples had collected into compact bands in the stacking gel portion, the gel was stopped and extracted and washed 3x for 5 minutes in Milli-Q water. The gel was stained in SimplyBlue SafeStain (Invitrogen, Cat. # LC6060) for 1 hour at RT. The gel was destained 2x for 1 hour in Milli-Q water. The gel bands were cut out and submitted to the mass spec core in microcentrifuge tubes filled with water for processing. pcDNA3-3x Myc-EV was used as the control sample in the NKX2.2 experiment and IgG was used as the control sample in the CHD4 experiment.

### NKX2.2 & CHD4 Mass Spectrometry

Samples were tryptically digested prior to LC/MS/MS analysis according to an established protocol (42, 43). Briefly, under keratin-free conditions, stacking gel bands were excised from the gel and dehydrated before being incubated with 25 ng/μL of trypsin at 37°C for 18 hrs, after which the enzyme was deactivated with 10% formic acid (FA). Peptides were extracted from the gel fragments, dried via speedvac, and resuspended for mass spectrometric analysis. NKX2.2 samples were re-suspended in 20 μL of buffer and CHD4 samples were re-suspended in 14 μL of buffer. NKX2.2 samples were analyzed similar to a previous publication (44), with the following exceptions: MS/MS data was collected in positive ion polarity over mass ranges 260–1700 m/z at a scan rate of 8 spectra/second for MS scans and mass ranges 50–1700 m/z at a scan rate of 3 spectra/second for MS/MS scans.

2 μL of CHD4 samples were acquired on an Orbitrap Eclipse (Thermo Scientific) operated using intensity-dependent CID MS/MS to generate peptide ID’s and equipped with a Ultimate 3000 RSCLnano LC system (Thermo Scientific) using a previously published method with the following exceptions: The orbitrap MS1 scan mass range was set to 375-1500 m/z range and dynamic exclusion was set to 20 seconds instead of 14 seconds (45). PEAKS studio proteomics version 10.5 was used to extract, search, and summarize peptide identity results from IgG and CHD4 samples (PEAKS, Waterloo, ON, Canada). Peptide identifications were performed using the PEAKS DB search engine combined with PEAKS de novo sequencing. Extracted spectra were searched against the SwissProt Mus musculus database. The crude PEAKS studio proteomics extractor protein abundance levels were used to compare relative abundances of proteins in each sample to determine protein interactors for CHD4.

### Co-Immunoprecipitation and Western Blotting

For Co-IP and western of endogenous CHD4, pulldowns were performed in WT MIN6 cells using the Nuclear Complex Co-IP Kit (Active Motif, Cat. #54001) per the manufacturer’s instructions. Briefly, 500 μg of WT MIN6 nuclear extract was incubated in IP High Buffer (no detergent and no NaCl) using 5 μg of CHD4 rabbit antibody (D4B7, CST, Cat. #12011) or 5 μg of a control IgG rabbit antibody (CST, Cat. #2729), overnight with mixing. The second day, samples were incubated with Anti-Protein-A magnetic beads (Pierce, Cat. #88846) for 1 hour with mixing at 4°C. Samples were then washed using the IP High Buffer (no detergent and no NaCl) with and without BSA. For all samples, including 5% input samples, 2x Laemmli buffer (Bio-rad, Cat. #1610737) plus βME was used as a sample buffer and samples were boiled for 5 minutes at 95°C and loaded in a Bio-Rad Mini-PROTEAN TGX Gel (4-20%, 10-well, 50 μL, Bio-rad, Cat. # 456-1094) with a Precision Plus Protein Ladder (Bio-rad, Cat. #1610375). Protein gels were transferred onto PVDF membranes and washed 3x for 5 minutes in TBS-T at RT. Membranes were blocked in TBS-T + 5% milk for 1 hour at room temp and membranes were washed 3x for 5 minutes in TBS-T at RT. Primary antibodies (Supplemental Table #2) were added at concentrations noted in TBS-T + 5% BSA overnight on a rocker at 4°C. The next day, membranes were washed 3x for 5 minutes in TBS-T at RT and an IP Veriblot HRP secondary antibody was added at 1:500 in TBS-T + 5% Milk (abcam, Cat. #ab131366). Membranes were washed 3x for 5 minutes in TBS-T at RT and imaged using SuperSignal West Pico PLUS Chemiluminescent Substrate (Thermo Scientific, Cat. #34580).

### Mouse Islet Western Blotting

For western blot of endogenous CHD4 in mouse islets, islets from 3 mice of control or mutant genotype were isolated as described above and were pooled to form 1 control or mutant sample. Islets were lysed using 1x RIPA Buffer (Abcam, Cat. # 156034) + PIC tablets (Invitrogen, Cat. # A32961) for 30 minutes on ice. Samples were spun down at 12,000 rpm for 20 minutes at 4°C and protein concentration was assessed using the DC assay kit (Bio-Rad, Cat. #5000112). 50 μg of Control or Mutant Mouse Islet Lysate was used and western was run as described above. Vinculin was used as the loading control (Supplemental Table #2).

### Generation of Chd4 KO Mice and Animal Maintenance

*Chd4* pancreas KO mutant mice were created by breeding *Pdx1^(Cre/+)^* mice (B6.FVB-Tg(Pdx1-cre)6Tuv/J) (23) and *Chd4^(flox/flox)^* (Chd4^tm1.1Kge^) created in the Georgopoulos lab (16). *Chd4* βKO mutant mice were created by breeding *Ins1^(Cre/+)^* mice (B6(Cg)-*Ins1^tm1.1(cre)Thor^*/J) (24) and *Chd4^(flox/flox)^* mice. For experiments requiring cell sorting, *Ins2^(GFP/+)^* reporter mice (29) were bred into the *Chd4* control and βKO line. Physiological studies were conducted on male and female mice with ages of mice being noted for each experiment. Mice were kept in accordance with University of Colorado Institutional Animal Care and Use Committee (IACUC) protocol #00045. Genotyping primers are listed in Supplemental Table #1.

### Blood Glucose Readings

Fasted blood glucose was measured after a 6 hour fast from mouse tail vein. *Ad libitum* blood glucose readings were taken from the mouse tail vein of fed mice. All glucose readings were taken using a Contour 7151H glucose meter and Contour 7097C test strips.

### Glucose Tolerance Test

Mice were fasted for 6 hours and initial blood glucose readings were taken at timepoint 0 from mouse tail vein. Mice were then injected with 2mg per gram of body weight D-glucose (Sigma, Cat. #G8270-1Kg). Additional tail vein blood glucose readings were taken at time 15, 30, 60, 90 and 120 minutes. All glucose readings were taken using a Contour 7151H glucose meter and Contour 7097C test strips. The area under the curve was analyzed using PrismGraphPad 10.4.1.

### Mouse Pancreas Tissue Preparation and Immunofluorescence Staining

Mouse pancreata were dissected at specified ages and fixed in 4% PFA for 4 hours. Following fixation, pancreata were placed in 30% sucrose overnight then embedded in Optimal Cutting Temperature (OCT) Compound (Sakura, Cat. #4583) and frozen at −80°C. Tissue blocks were sectioned using a cryostat (Thermo Microm525) at a slice thickness of 10μm. Three consecutive slices were added to each slide until the entire pancreas was sectioned completely through, around 100-120 slides total.

For immunofluorescent staining, every 10^th^ slide, covering the entire pancreas was stained. Prior to staining, slides were thawed for 10 minutes. If antigen retrieval was necessary slides were preheated with Na Citrate (10mM Na Citrate, 0.05% Tween, pH 6.00) and heated for 20 minutes followed by washes using PBS-T (0.1% Tween-20). Slides were then blocked using PBS-T + 2% Normal Donkey Serum (NDS) at room temperature (RT) for 30 minutes. Slides were washed 1x in PBS-T for 5 minutes at RT followed by addition of primary antibodies (Supplemental Table #2). Slides were incubated overnight at 4 degrees in humid chamber. The following day slides were washed 4x in PBS-T for 5 minutes at RT followed by addition of secondary antibodies (1:500 in PBS-T + 2% NDS). Secondary antibodies were incubated on slides for 1-3 hours in humid chamber at RT. Slides were washed 4x in PBS-T for 5 minutes at RT followed by addition of DAPI (1:1000 in PBS-T) and incubated for 15 minutes at RT. Slides were washed 2x in PBS-T for 5 minutes at RT followed by addition of Prolong Gold mounting reagent (Invitrogen P36930), 22x60 cover slips and sealed using clear nail polish (Electron Microscopy Sciences, Cat. #72810).

### Image Analysis and Quantification

For the P2, 3 week and 6-week timepoints, images were taken on a Leica DM5500B with a Leica DFC 345 FX and Leica DFC425 camera. Filters cubes were A4, L5 and N3. For the P2 timepoint, pictures of entire pancreas sections were taken using the tilescan function using the 10x objective. For the 3-week and 6-week timepoints, pictures of all individual islets were taken using a 20x objective from one of the three pancreas slices of each slide stained. Each islet immunofluorescent image was quantified using ImageJ2 Version 2.14.0/1.54f. Full islet area was quantified as well as positive hormone area by quantifying all area above a set threshold (50 for all channels). Hormone area for P2 was normalized by total pancreas area and hormone area for 3- and 6-week timepoints was normalized by total islet area. For all other immunofluorescent staining images, images were taken on a Zeiss LSM 800 with the objective specified and using a 405, 488, 561 and 640 nm laser line.

### Whole Mouse Pancreas Acid Ethanol Insulin Collection

10-20 mg sections of whole mouse pancreatic tissue were removed from both controls and *Chd4* βKO mutant mice and placed in 1 mL of 0.18M HCl in 70% EtOH (acid ethanol) and sonicated 2 x 30 seconds (Fisher Scientific Cat. #FS30H) and vortexed for 1 minute. Whole pancreas tissue was incubated at 4°C for 12 hours and stored at −80°C until ELISAs were performed using Mercodia Insulin ELISA (Cat. #10-1247-01) or Mercodia ProInsulin ELISA (Cat. #10-1232-01) per the manufacturer’s instructions.

### Generation of Full Mouse Pancreas Slices

Pancreas tissue slices were prepared as previously described (28). Briefly, animals were injected with 100 mg/kg ketamine and 8 mg/kg xylazine and euthanized via exsanguination prior to inflating the pancreas with low-melting-point 1.25% agarose solution dissolved in extracellular solution (ECS, consisting in 140 mM NaCl, 5 mM KCl, 2 mM NaHCO_3_, 1 mM NaH_2_PO_4_, 1.2 mM MgCl_2_, 1.5 mM CaCl_2_, 3 mM glucose and 10 mM HEPES [pH is adjusted to 7.4 with NaOH]) at 37°C into the common bile duct. Immediately after injection, the agarose infused pancreas was cooled with ice-cold ECS, excised, trimmed into smaller blocks and embedded in 1.25% agarose. Tissues were sliced with a thickness of 200 μm with a vibratome (VF-310-0Z, Precisionary) and collected in ice-cold ECS. Slices were then placed in a 24-well plate containing 1 ml/well of ECS with Soybean Trypsin (Final concentration 0.1 mg/ml) inhibitor to prevent any inadvertent degradation by digestive enzymes. Slices were washed on an orbital shaker at 4°C for 1-hour prior to selection of islet containing slices. Slices were then placed in cell culture inserts coated with a collagen mixture (28) in a 6-well plate with 2 ml of RPMI medium supplemented with 10% FBS, 1% Pen-Strep, and trypsin inhibitor (Final Concentration 0.1 mg/ml), and kept overnight in the Incubator at 37°C and 5% CO_2_. Slices were then used to perform Glucose Stimulated Insulin Secretion (GSIS) and intracellular calcium imaging experiments.

### Mouse Pancreas Slices Glucose Stimulated Hormone Secretion Assays and Analysis

Insulin was assayed using static hormone secretion assays. All solutions were prepared in Krebs-Ringer buffer (108.8 mM NaCl, 5 mM NaHCO_3_, 5.8 mM KCl, 1.2 mM KH_2_PO_4_, 2.5 mM CaCl_2_, 1.2 mM MgSO_4_, 10 mM HEPES, 0.1% BSA, pH 7.4) and supplemented with either 2mM, 11mM, or 20 mM Glucose and 20mM, 11 mM, or 2 mM NaCl for osmotic balance. Duplicate sets of 3 or 5 pancreatic slices per condition were incubated in 2 mL of Krebs-Ringer buffer containing 2 mM glucose for 1 hour to establish baseline secretion. Then, each slice set was placed under a sequential 30-minute stimulation condition under one of three conditions: (1) 2 mM glucose, (2) 11 mM glucose, or (3) 20 mM glucose. A final 30-minute stimulation was performed with 20 mM + KCl in addition to each respective glucose/NaCl condition. After each 30-minute stimulation, 500 μL of supernatant was collected for analysis. Samples were stored at −20°C until analysis. Insulin concentrations were measured using a mouse ultrasensitive insulin ELISA kit (Crystal Chem, Cat. #90096) per the manufacturer’s instructions.

### Mouse Islet Isolation and Dissociation

Islets were isolated by the Islet Isolation Core facility. Briefly, mice were anesthetized using a ketamine/xylazine/acepromazine (KXA) mixture and euthanized by exsanguination. The pancreatic duct was clamped off and a 26-gauge needle with a collagenase mixture was injected into the pancreas. The pancreas was dissected out of the mouse and islets were isolated by a density gradient purification step followed by hand picking.

After isolation, islets were collected and placed in a 15mL tube and allowed to settle for 20 minutes. Islets were washed with pre-warmed PBS and spun at 300 x g for 3 minutes at RT. PBS was aspirated off and islets were resuspended in 2 mL of Accutase (Stemcell Tech, Cat. #07920) and placed in a 37°C water bath. Islets were incubated for 30 minutes total, resuspending islets by pipetting every 5 minutes. Accutase dissociation was stopped by adding RPMI1640 (Gibco, Cat. #11875-093) + 10% FBS. Dissociated islet cells were filtered through a 40μm cell strainer (Greiner, Cat. #542040) into a 50mL conical tube. Cells were spun at 300 x g for 3 minutes at RT. Supernatant was carefully aspirated off without disturbing cell pellet and cells were washed in 10mL FACS buffer (PBS + 1mM EDTA + 0.2% BSA). Cells were spun at 300 x g for 3 minutes at RT, supernatant was aspirated off and cells were resuspended in 1mL of FACS buffer for sorting. Propidium Iodide (PI, Thermo Cat. #P3566) was added as a live/dead marker at 1:1000.

### Mouse Islet β Cell Fluorescent Activated Cell Sorting (FACS)

β cells were sorted from dissociated 4 week mouse islets using an *Ins2^(GFP/+)^* reporter (29) on a Bio-rad S3e cell sorter. Gates were set to initially read FSC and SSC area to sort out debris and cell doublets. Cells were then gated using a PI negative to separate out living vs dead cells and lastly cells were gated using a GFP positive gate to sort and collect all β cells (Supplementary Figure 5). Cells were collected in 350 μL of RLT Lysis buffer + βME (Qiagen RNeasy Micro Kit, Cat. #74004), prior to RNA extraction.

### RNA Extraction and RNA Sequencing

RNA extraction was performed using the RNeasy Micro Kit (Qiagen, Cat. 74004) protocol. 2-Mercaptoethanol (βME) was added to RLT lysis buffer to help inhibit RNases. RNA quality was assessed using an Agilent 4200 TapeStation System and RNA samples with RNA Integrity Numbers (RIN) scores of 8 or higher were used for sequencing. RNA samples were sequenced at the Genomics Core facility on the University of Colorado Anschutz Medical Campus. Paired end libraries were made by the core from low input ribo-depleted RNA using SMARTer Stranded Total RNA-Seq Kit v2 – Pico Input Mammalian (Takara Cat.# 634411). All libraries were run on a NovaSEQ X machine at a sequencing depth of 50 million read pairs / 100 million total reads per sample. Fastq files were quality checked using FastQC (v0.12.1) (46) and adapters were trimmed using Cutadapt (v4.8) (47). The reads were mapped to the mm10 genome using STAR (v2.7.11a) (48). Counts tables were made using featureCounts (v2.0.6) (49) and used for differential gene expression analysis. Differential genes were assessed using DESeq2 (v1.46.0) (50) and significant genes were established using a padj value of ≤ 0.05 and a Log_2_ Fold Change value of > 0.5 or < 0.5.

### Reanalysis of ATAC-seq Dataset

The reanalysis of the published ATAC-seq data (22) was conducted by downloading Fastq files from the GEO database under reference number GSE217444. Files were processed using our bulk ATAC-seq snakemake pipeline. Briefly, Fastq files were quality checked using FastQC (v0.12.1) (46) and adapters were trimmed using Cutadapt (v4.8) (47). The reads were aligned to the mm10 genome using Bowtie2 (v2.5.3) (51) and mitochondrial reads were removed using Samtools (v1.20) (52). The ATACseqQC package (v1.28) (53) was used to generate final quality metric plots, and FRiP values were tabulated using featureCounts (v2.0.6) (49). We used MACS3 (v3.0.2) (54) with a q-value cutoff of 0.01 to call peaks within the dataset, then utilized DiffBind (v3.14) (55) with DESeq2 (v1.46.0) (50) and a padj value < 0.05 to identify differentially accessible peaks between the CHD4 Control and CHD4 βKO ATAC-seqs.

### CHD4 ChIP-qPCR

20 million MIN6 cells were collected for use in the SimpleChIP Enzymatic Chromatin IP Kit (Magnetic Beads, CST, Cat. #9003) per the manufacturer’s instructions. Briefly, cells were crosslinked with 1% formaldehyde for 10 minutes and crosslinking was quenched with glycine. Cells were lysed and chromatin was fragmented using a Diagenode Bioruptor Pico sonicator and Micrococcal Nuclease (CST, Cat. # 10011) to fragment chromatin to approximately 150-900bp. 2% of samples was removed to be used as input before pulling down CHD4. 30 μg of fragmented chromatin DNA was used for IP with 10 μL CHD4 antibody (Rabbit, CST D4B7, 12011S) or 2μL IgG (Rabbit, CST, Cat. #2729). Samples were next incubated overnight with rotation at 4°C and 30 μL of ChIP-Grade Protein G Magnetic Beads (CST, Cat. #9006) was added, followed by incubating samples again for 2 hours with rotation at 4°C. Chromatin was eluted from beads and cross-links were reversed using NaCl, RNase A and Proteinase K and DNA was purified from samples using DNA spin columns.

For qPCR, 3 replicates of 5 ng of CHD4 IP or IgG IP samples were used with iTaq Universal SYBR Green Supermix (Bio-Rad, Cat. #1725120) and primers (Supplementary Table #1). A Bio-Rad CFX96 system was used to run qPCR plate. Fold enrichment over IgG was used to quantify CHD4 binding over IgG control.

### Mouse Pancreas Slices Calcium Signaling and Analysis

Slices were incubated with 4 μM Fluo-4 AM (Thermo Fisher Scientific Cat. #F14201) in BMHH buffer containing 125 mM NaCl, 5.7 mM KCl, 2.5 mM CaCl_2_, 1.2 mM MgCl_2_, 10 mM HEPES, 0.1% bovine serum albumin (BSA), 2 mM glucose, and 1mg/ml Trypsin inhibitor at room temperature for 30 min in the dark. Slices were transferred to MatTek glass-bottom imaging dishes (MatTek, Cat. #P35G-1.5-14-C) with fresh BMHH with 2 mM glucose prior to imaging. An anchor was placed to hold the slice in place. Slices were imaged on a Zeiss laser scanning microscope (LSM) 800 with a 40x objective using a 488 nm laser. Slices were imaged at 37°C, where images were acquired every second for 2.5–5 min at 2mM glucose. Following imaging, medium was changed to 11 mM glucose and images were acquired for another 15-30 min. Fiji was used to extrapolate the calcium time courses. The time courses were normalized for intensity and normalized for movement if needed. The area under the curve was analyzed using PrismGraphPad 10.4.1.

### Statistical Analysis

Unless otherwise stated, error bars are shown as mean +/− standard deviation (SD) and Shapiro-Wilk test was used to test normality. If passed, unpaired two-tailed student’s t-test was used to determine significant differences, otherwise, Mann-Whitney U test was used. P-value or padj of ≤ 0.05 was used to determine statistical significance. If no statistics are shown on graphs, results were not significant.

## Supplementary Material

Supplement 1

## Data Availability

ATAC-sequencing was reanalyzed from a previously published study (GSE217444). Code for the analysis of the sequencing data is available on Github (https://github.com/CUAnschutzBDC/snakemake_pipelines/tree/6ea22de25f49706bb268502bbd894a4a4b7cdb55/RNA_seq, https://github.com/CUAnschutzBDC/snakemake_pipelines/tree/6ea22de25f49706bb268502bbd894a4a4b7cdb55/ATAC_seq) and docker containers for the RNA-seq and ATAC-seq are available on docker hub (RNA-seq docker: https://hub.docker.com/layers/kwellswrasman/rnaseq_general/v1/images/sha256-2536b14130bba19b70f28d23807f127854cd646bbc4bd4028407eeb35ffea45f, https://hub.docker.com/layers/kwellswrasman/rnaseq_r/v1/images/sha256-36d320d7de293da2a84ff35ab131ef6ebec42ab53934aacc9d4f341f7da33988. ATAC-seq docker: https://hub.docker.com/layers/kwellswrasman/fastq_screen/v1/images/sha256-cf697c13d436f251dc70048adfee58438576698c03e398455c394c2d16ffac59, https://hub.docker.com/layers/kwellswrasman/general_chip/v2/images/sha256-447cd6c6ab57006b75b285f851443b9b9d0bc06c8d882bb1a1cb3387d61cc499, https://hub.docker.com/layers/kwellswrasman/atac_chip_r/v1/images/sha256-e499fc2bcde315a720a442447decb95c3a4fa084466bdf816e164f2e40f01971, https://hub.docker.com/repository/docker/kwellswrasman/picard/tags/v1/sha256-29984d83a5ada3c1799684c36bfb4a1dce6912a105baa3b87828b3ef939ee7de).
