# Supplementary material for: The Chromatin Remodeler Protein CHD4 Cooperates With NKX2.2 to Regulate Pancreatic Beta Cell Integrity
Source: bioRxiv. 2025 Jun 17:2025.06.16.659956. Preprint. [Version 1] doi: 10.1101/2025.06.16.659956 (PMC12262308; doi:10.1101/2025.06.16.659956)
Supplement: Supplementary file 1 [file media-1.pdf]

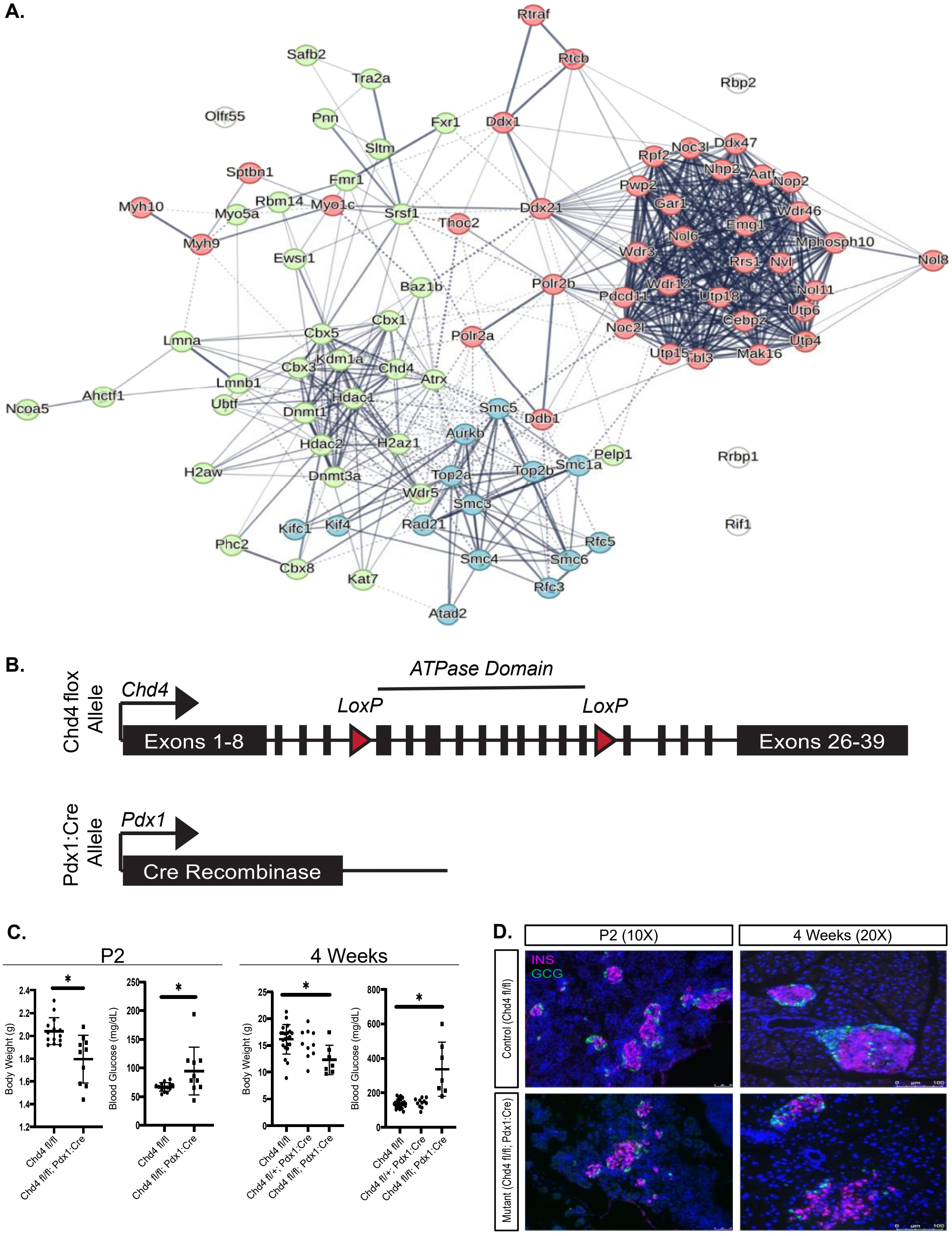
A.
Rtraf
Rtcb
Safb2
Tra2a
Rbp2
Olfr55
Pnn
Fxr1
Ddx1
Sltm
Noc3l
Ddx47
Rpf2
Sptbn1
Fmr1
Nhp2
Aatf
Nop2
Pwp2
Rbm14
Myo1c
Gar1
Myh10
Myo5a
Srsf1
Ddx21
Wdr46
Thoc2
Emg1
Nol6
Myh9
Mphosph10
Wdr3
Rrs1
Nvl
Nol8
Ewsr1
Baz1b
Polr2b
Wdr12
Pdcd11
Utp18
Nol11
Utp6
Cbx1
Cebpz
Cbx5
Polr2a
Noc2l
Lmna
Utp4
Kdm1a
Utp15
Tbl3
Mak16
Cbx3
Chd4
Atrx
Lmnb1
Ahctf1
Hdac1
Ubtf
Ncoa5
Smc5
Dnmt1
Ddb1
Aurkb
Hdac2
H2az1
Pelp1
Rrbp1
Smc1a
Top2b
Top2a
H2aw
Dnmt3a
Wdr5
Smc3
Rif1
Kifc1
Kif4
Rad21
Rfc5
Phc2
Smc4
Smc6
Cbx8
Kat7
Rfc3
Atad2
B.
ATPase Domain
Chd4 flox Allele
Chd4
LoxP
LoxP
Exons 1-8
Exons 26-39
Pdx1:Cre Allele
Pdx1
Cre Recombinase
C.
P2
4 Weeks
Body Weight (g)
Blood Glucose (mg/dL)
Body Weight (g)
Blood Glucose (mg/dL)
Chd4 fl/fl
Chd4 fl/fl; Pdx1:Cre
Chd4 fl/+; Pdx1:Cre
D.
P2 (10X)
4 Weeks (20X)
INS
GCG
Control (Chd4 fl/fl)
Mutant (Chd4 fl/fl; Pdx1:Cre)

Supplementary Figure 1.

Deletion of *Chd4* from the developing pancreas causes hyperglycemia and body weight defects. (A) String database clustering identified 112 overlapping proteins from NKX2.2 IP-MS and CHD4 IP-MS that grouped into 3 k-means clusters (20). (B) Schematic of Pancreas and Duodenum *Chd4* KO allele. Flanking Lox P (flox) sites are located upstream of exon 12 and downstream of exon 21, excising the ATPase domain. (C) Measurements of body weight and *ad libitum* blood glucose for P2 control ($Chd4^{fl/fl}$, n =14) and mutant ($Chd4^{fl/fl}$; $Pdx1^{Cre/+}$, n=10) mice (two-tailed Student's *t*-test). Measurements of body weight and *Ad libitum* blood glucose for 4-week control ($Chd4^{fl/fl}$, n=22), Het ($Chd4^{fl/+}$; $Pdx1^{Cre/+}$, n=10) and mutant ($Chd4^{fl/fl}$; $Pdx1^{Cre/+}$, n=7) mice (one-way ANOVA test). (D) Representative immunofluorescent images for P2 and 4-week control and mutant mice showing DAPI (blue), Insulin (INS, magenta), and Glucagon (GCG, green). P2 IF images are at 10x magnification with Scale bars: 50 µm. 4-week IF images are at 20x magnification with Scale bars: 100 µm. *$P \leq 0.05$.

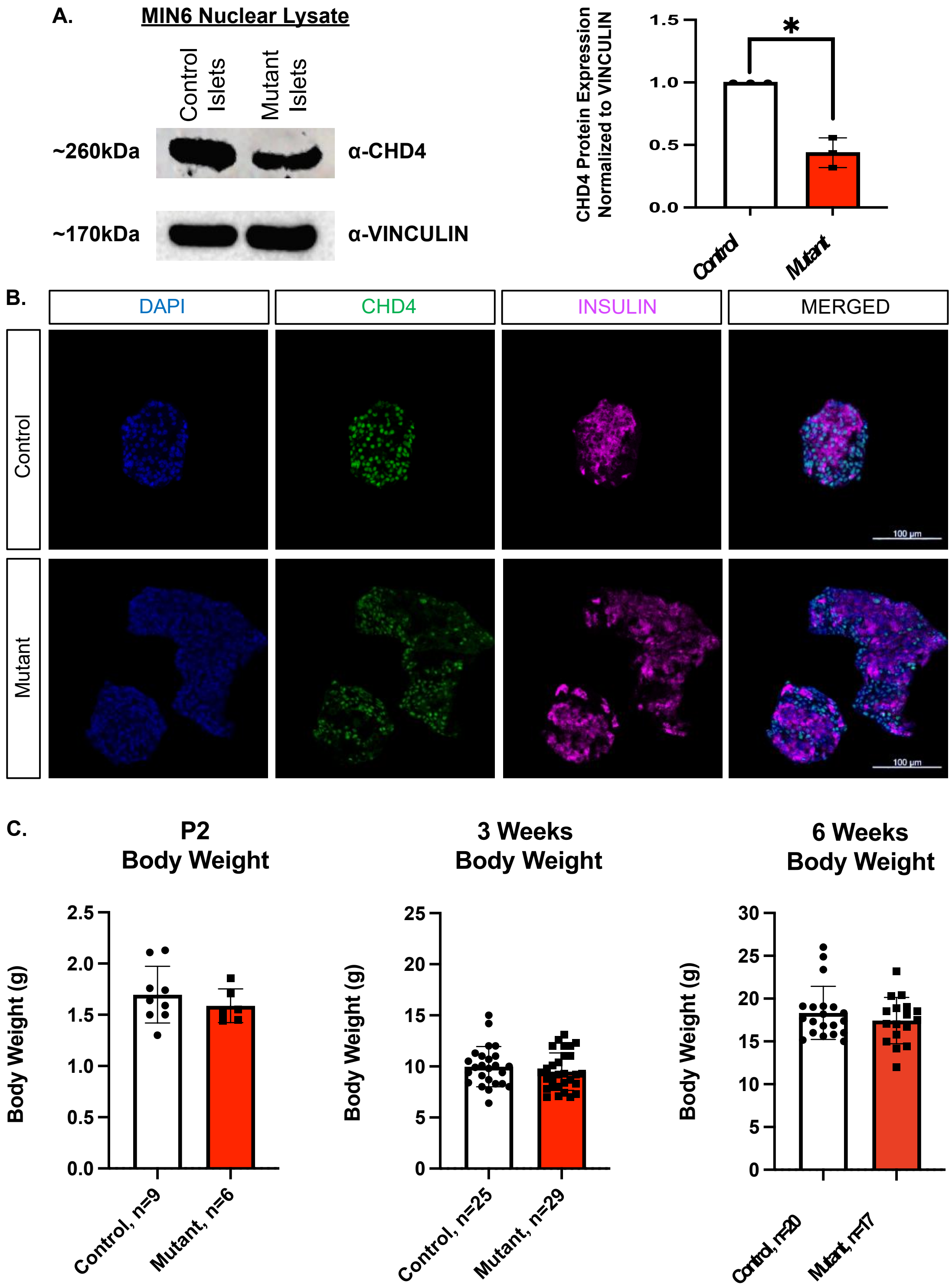
A.
MIN6 Nuclear Lysate
Control Islets
Mutant Islets
~260kDa
α-CHD4
~170kDa
α-VINCULIN
CHD4 Protein Expression Normalized to VINCULIN
1.5
1.0
0.5
0.0
*
Control
Mutant
B.
DAPI
CHD4
INSULIN
MERGED
Control
Mutant
100 µm
C.
P2 Body Weight
3 Weeks Body Weight
6 Weeks Body Weight
Body Weight (g)
2.5
2.0
1.5
1.0
0.5
0.0
Control, n=9
Mutant, n=6
25
20
15
10
5
0
Control, n=25
Mutant, n=29
30
25
20
15
10
5
0
Control, n=20
Mutant, n=17

Supplementary Figure 2.

***Chd4* is efficiently deleted from mouse islet β cells without a disruption in body weight.** (A) Western blot analysis of CHD4 expression in mouse islet lysate. Quantification of CHD4 expression normalized to Vinculin between control and mutant lysates (two-tailed Student's *t*-test). (B) Representative immunofluorescent images for 3-week control and mutant mice showing *Chd4* KO. DAPI (blue), Insulin (magenta), and CHD4 (green). Scale bars: 100 μm. (C) Measurements of body weight for P2, control (n=9) and mutant (n=6*)* mice (two-tailed Student's *t*-test). Measurements of body weight for 3-week, control (n =25) and mutant (n=29*)* mice (two-tailed Student's *t*-test). Measurements of body weight for 6-week, control (n =20) and mutant (n=17*)* mice (Mann Whitney U test). *$P \leq 0.05$.

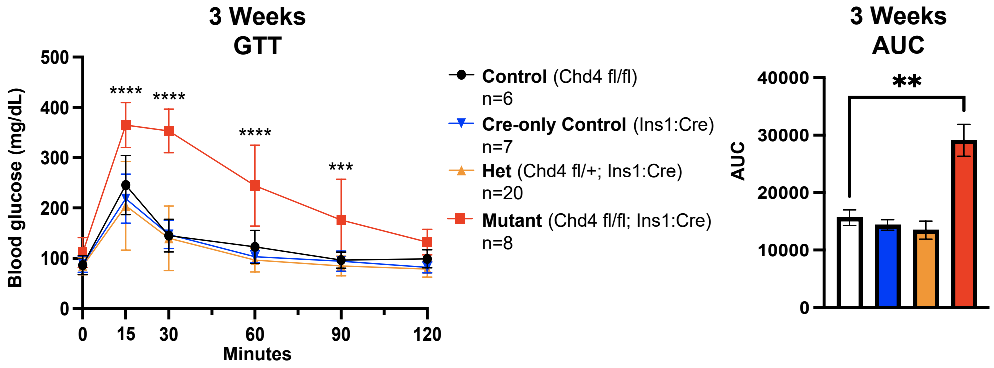

Supplementary Figure 3.

All control genotypes have similar fasted blood glucose and glucose tolerance levels.
3-week combined sex Glucose Tolerance Test (GTT) of control (*Chd4*$^{fl/fl}$: black line, n=6), Cre-only control (*Ins1*$^{Cre/+}$: blue line, n=7), het (*Chd4*$^{fl/+}$*; Ins1*$^{Cre/+}$: orange line, n=20), and mutant (*Chd4*$^{fl/fl}$*; Ins1*$^{Cre/+}$: red line, n=8) mice (multiple comparison with 2 way ANOVA). Area under the curve (AUC) measurement for respective 3-week GTT. All GTT points normalized to 0 minutes time point prior to AUC calculation (one-way ANOVA). ***P*≤0.01, ****P*≤0.001, *****P*≤0.0001.

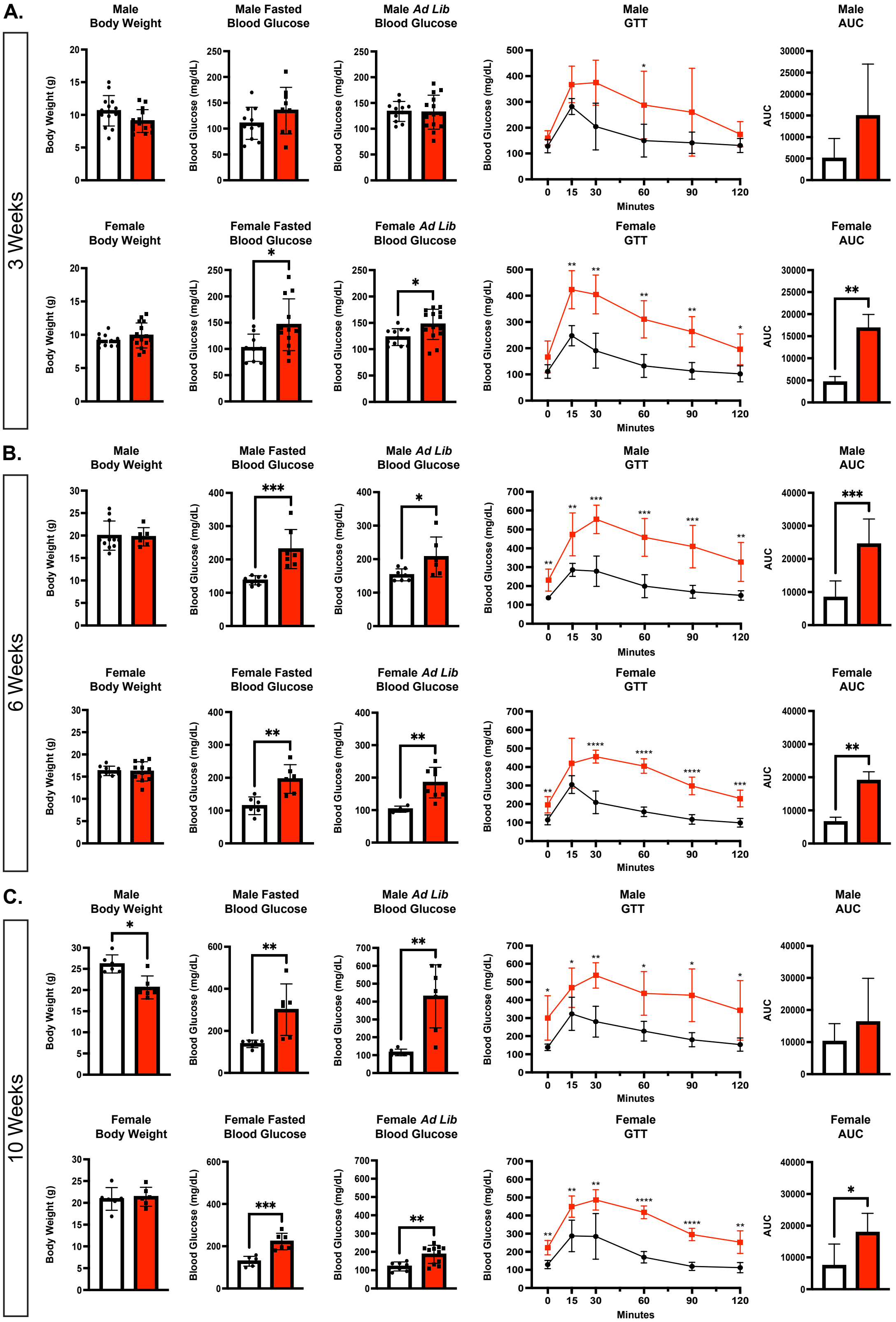
A.
B.
C.
3 Weeks
6 Weeks
10 Weeks
Male Body Weight
Male Fasted Blood Glucose
Male *Ad Lib* Blood Glucose
Male GTT
Male AUC
Female Body Weight
Female Fasted Blood Glucose
Female *Ad Lib* Blood Glucose
Female GTT
Female AUC
Body Weight (g)
Blood Glucose (mg/dL)
AUC
Minutes

Supplementary Figure 4.

Both male and female *Chd4* βKO mice show a worsening diabetic phenotype.

Unless otherwise noted, mouse data consists of control (*Chd4*$^{fl/fl}$: white Bars) and mutant/*Chd4* βKO (*Chd4*$^{fl/fl}$*; Ins1*$^{Cre/+}$: red Bars) genotypes. (A) Measurements of 3-week male control (n =14) and mutant (n=14*)* body weight. 3-week female control (n =11) and mutant (n=15*)* body weight. 3-week male control (n =11) and mutant (n=9*)* fasted blood glucose. 3-week female control (n =9) and mutant (n=12*)* fasted blood glucose. 3-week male control (n =11) and mutant (n=14*) Ad libitum (Ad Lib)* blood glucose. 3-week female control (n =11) and mutant (n=14*) Ad Lib* blood glucose. 3-week male Glucose Tolerance Test (GTT) of control (black line, n=6) and mutant (red line, n=6) mice. 3-week female GTT of control (black line, n=6) and mutant (red line, n=6) mice. Area under the curve (AUC) measurement for respective 3-week GTTs. All points normalized to 0 minutes time point prior to AUC calculation. (B) Measurements of 6-week male control (n =11) and mutant (n=6*)* body weight. 6-week female control (n =9) and mutant (n=11*)* body weight. 6-week male control (n =7) and mutant (n=7*)* fasted blood glucose. 6-week female control (n =6) and mutant (n=6*)* fasted blood glucose. 6-week male control (n =7) and mutant (n=6*) Ad Lib* blood glucose. 6-week female control (n =4) and mutant (n=8*) Ad Lib* blood glucose. 6-week male GTT of Control (black line, n=7) and mutant (red line, n=7) mice. 6-week female GTT of control (black line, n=6) and mutant (red line, n=6) mice. Area under the curve (AUC) measurement for respective 6-week GTTs. All points normalized to 0 minutes time point prior to AUC calculation. All points normalized to 0 minutes time point prior to AUC calculation. (C) Measurements of 10-week male control (n =6) and mutant (n=6*)* body weight. 10-week female control (n =6) and mutant (n=6*)* body weight. 10-week male control (n =6) and mutant (n=6*)* fasted blood glucose. 10-week female control (n =6) and mutant (n=6*)* fasted blood glucose. 10-week male control (n =6) and mutant (n=7*) Ad Lib* blood glucose. 10-week female control (n =7) and mutant (n=11*) Ad Lib* blood glucose. 10-week male GTT of control (black line, n=6) and mutant (red line, n=6) mice. 6-week female GTT of control (black line, n=6) and mutant (red line, n=6) mice. Area under the curve (AUC) measurement for respective 10-week GTTs. All points normalized to 0 minutes time point prior to AUC calculation. For all figures, *$P \leq 0.05$, **$P \leq 0.01$, ***$P \leq 0.001$, ****$P \leq 0.0001$. For GTT figures, multiple two-tailed Student's *t*-tests were used. For all other figures, two-tailed Student's *t*-test except Male 6-week body weight, Male 6-week fasted blood glucose, Male 10-week body weight, and Male 10-week fasted blood glucose where Mann Whitney U tests were used.

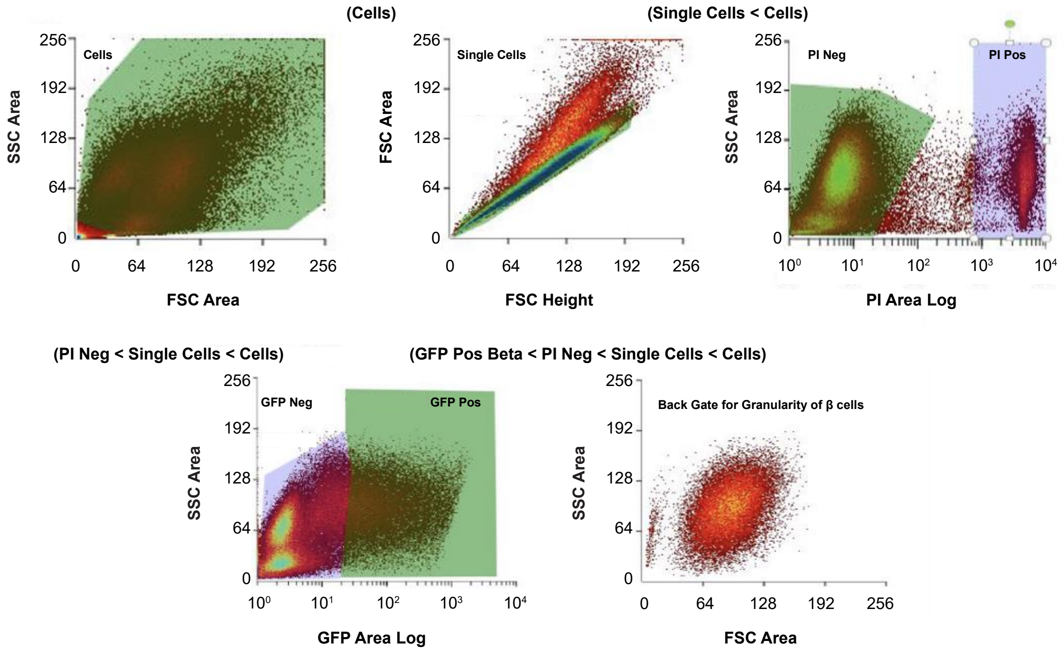

Supplementary Figure 5.
Gating used to sort β cells using GFP. β cells were sorted using an *Ins2*$^{(GFP/+)}$ reporter allele, bred into control and mutant mice. Cells were initially sorted using FSC area and SSC area to remove debris and doublet cells. Cells were next sorted using propidium iodide (PI) as a live/dead marker, gating on live cells. Cells were next sorted using a GFP+ area. Lastly, cells were back gated to FSC area and SSC area to check granularity of cells to make sure they replicate β cell granularity.

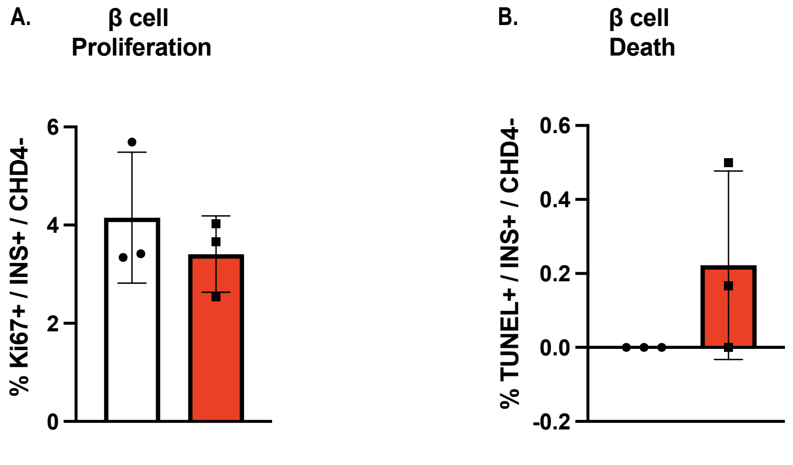

Supplementary Figure 6.
β cell proliferation and TUNEL staining results. (A) Quantification of P2 proliferation using Ki-67 immunofluorescence staining. Control (n=3) and mutant (n=3) (two-tailed Student's *t*-test). (B) Quantification of 3-week cell death using TUNEL immunofluorescence staining. Control (n=3) and mutant (n=3) (two-tailed Student's *t*-test).

PCR Genotyping Primer Sequences	
**Target**	**Sequence**
Chd4 Flox FWD	CTC CAG AAG AAG ACG GCA GAT CT
Chd4 Flox REV	GTC CTT CCA AGA GAG AGC AAG
Pdx1 CRE FWD	CCT GGA CTA CAT CTT GAG TTG C
Pdx1 CRE REV	AGG CAA ATT TTG GTG TAC GG
INS1 WT FWD	GTC AAA CAG CAT CTT TGT GGT C
INS1 CRE FWD	GCT GGA AGA TGG CGA TTA GC
INS1 REV	GGA AGC AGA ATT CCA GAT ACT TG
INS2 WT FWD	CAT CAG CAA GCA GGA AGG TA
INS2 GFP REV	CGG ACA CGC TGA ACT TGT G
**qPCR SYBR Primer Sequences**	
**Target**	**Sequence**
MafA FWD	GCC ATT TAC CAA GCC CCA AA
MafA REV	TGA GGA GCA AGC ACA GCA
Slc2a2 FWD	AGC CAC TGA GTT GTT GGG AA
Slc2a2 REV	GGG CTC TAA CAT GCT CTC CA
Kcnj5 FWD	CTG CAG TAC ACA GGA CAG C
Kcnj5 REV	CAC TCG TGA CAG ATT GCA GG

Supplementary Table 1.
PCR Genotyping and qPCR primer sequences

Target Antigen	Primary Host	Experiment	Company / Catalog #	Concentration
CHD4	Mouse	IF	Abcam / ab70469	1:250
INSULIN	Guinea Pig	IF	Agilent / IR00261-2	1:5
GLUCAGON	Rabbit	IF	CST / 2760	1:250
SOMATOSTATIN	Rabbit	IF	Pheonix / H-060-03	1:500
Ki-67	Rabbit	IF	Abcam / ab15580	1:500
TUNEL	TMR Red	TUNEL	Sigma / 12156792910	Per kit instructions
CHD4	Rabbit	IP / Western	CST / 11912	5 µg / 1:1000
CHD4	Rabbit	ChIP / Western	CST / 12011	10 µg / 1:1000
NKX2.2	Mouse	Western	DSHB / 74.5A5	.5 µg/mL
NKX2.2	Rabbit	Western	Sigma / HPA003468	1:250
MYC	Rabbit	IP / Western	Santa Cruz / sc-789	5 µg / 1:500
IgG	Rabbit	ChIP / IP	CST / 2729	5 µg or 10 µg
VINCULIN	Rabbit	Western	CST / 13901	1:500

Supplementary Table 2.
Experimental Antibodies

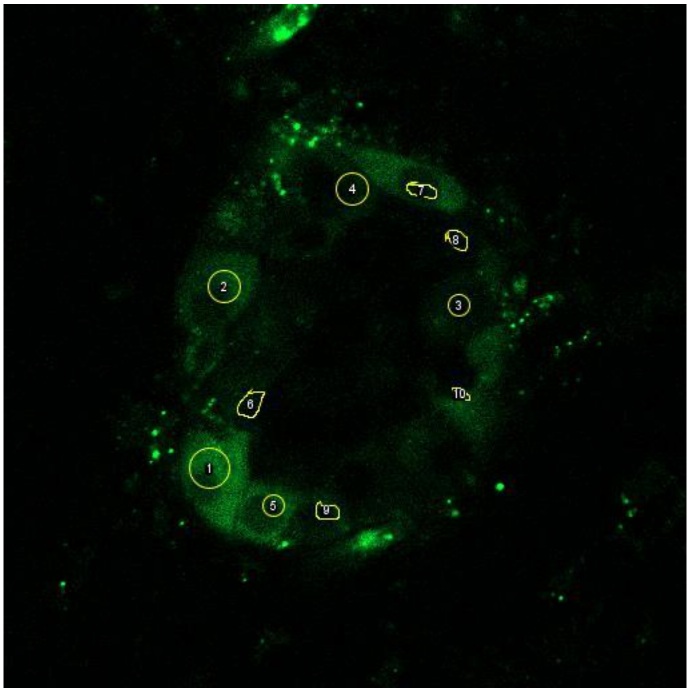

Supplementary Movie 1.

Control pancreas slices have functional calcium imaging.
Control pancreas slice calcium imaging shows responsive islet cells. Cells are numbered 1-10. At 3 seconds in video 11mM Calcium is applied to medium. Fluorescence is from Fluo-4 calcium reactive molecule.

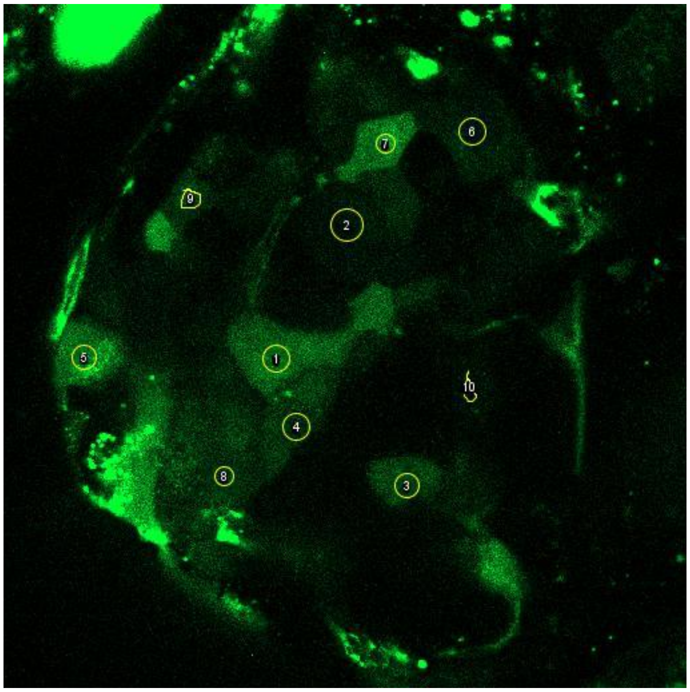

Supplementary Movie 2.

Mutant pancreas slices have dysfunctional calcium imaging.

Mutant pancreas slice calcium imaging shows non-responsive islet cells. Cells are numbered 1-9. At 3 seconds in video 11mM Calcium is applied to medium. Fluorescence is from Fluo-4 calcium reactive molecule.
